# Does radial shockwave therapy lead to immediate improvements in pain in people with insertional Achilles tendinopathy? A randomised controlled trial

**DOI:** 10.1177/02692155251394951

**Published:** 2025-11-27

**Authors:** B Alsulaimani, L Perraton, J Bourke, T Powers, P Malliaras

**Affiliations:** 1 Department of Physiotherapy, 22457 School of Primary and Allied Health Care, Monash University, Clayton, VIC, Australia; 2 Department of Physical Therapy, 2541 King Abdulaziz University, Jeddah, Saudi Arabia; 3 Monash eResearch Centre, 2541 Monash University, Clayton, VIC, Australia

**Keywords:** Insertional Achilles tendinopathy, shockwave, placebo

## Abstract

**Objectives:**

To investigate the immediate effects of radial shockwave therapy versus sham on movement evoked pain in people with insertional Achilles tendinopathy.

**Design:**

Randomised controlled trial.

**Setting:**

Private clinic.

**Participants:**

People diagnosed with insertional Achilles tendinopathy who were over 18 years old with a symptom duration of greater than 3 months.

**Intervention:**

Seventy-six participants (53% female, mean age 51 years) were randomly allocated to a radial shockwave (n = 38) or sham (n = 38) group. Three sessions of radial shockwave or sham (no pressure) to the most affected side in 5-to-10-day intervals. All participants received identical education and exercises.

**Main measures:**

The primary outcome measure was movement evoked pain (measured on a 100 mm visual analogue scale) at the first, second and third session immediately after each application.

**Results:**

There was 96% follow up of participants at the third session. Over half of the participants believed they were receiving the ‘real’ treatment (average 58%). The mean movement evoked pain scores improved each session by 0.6 points for the radial shockwave therapy and 0.7 points for the sham group. There was no difference between the groups after the first (−0.4, 95% confidence interval (CI) −1.6 to 0.8), second (0.4, 95% CI −0.8 to 1.6) or third session (−0.4, 95% CI −1.6 to 0.8).

**Conclusions:**

In adults with insertional Achilles tendinopathy, radial shockwave therapy demonstrated no more efficacy than a sham in reducing immediate movement evoked pain. These results do not support the use of radial shockwave therapy for immediate pain relief among people with this condition.

**Clinical trial registration:**

ACTRN12620000035921.

## Introduction

Insertional Achilles tendinopathy is a painful disorder that is characterised by localised load-related pain at the insertion of the Achilles tendon into the calcaneus.^1–3^ Exercise is a recommended first-line treatment, but it is estimated that 68% of people may not respond to exercise.^4^ A common adjunct therapy for insertional Achilles tendinopathy is radial shockwave therapy, which involves high frequency pressure waves delivered to the targeted tendon or tissue.^5^ There is some evidence for effectiveness when compared with exercise for a range of outcomes at medium term.^6^

It has been observed that there may be immediate pain-relieving effects of radial shockwave therapy.^7,8^ This could be explained by reduced local levels of substance P altering pain perception observed in animal model^9^ or conditioned pain modulation. Radial shockwave therapy is a painful treatment which may trigger endogenous pain inhibitory pathways in the same way as conditioned pain modulation laboratory procedures, such as the cold pressor test.^5,10^ A hallmark of conditioned pain modulation is immediate increase in pain tolerance to pressure at the local site of the painful stimulus and at remote sites.^11^ Understanding if changes in pain immediately follow radial shockwave therapy and if this is coupled with changes in pressure pain tolerance locally and remotely will inform the mechanism of action and support the use of this treatment clinically.

Only two trials thus far have investigated the efficacy of radial shockwave therapy and exercise compared to a sham intervention.^12,13^ Both trials used comparable interventions and found no between-group differences in the medium-term (12–24 weeks).^12,13^ However, there were limitations to these trials, including (i) neither trial assessed the immediate improvements or (ii) investigated the mechanism of action. Despite that it appears that radial shockwave therapy does not have a sustained effect for this condition in the medium term, it is currently unknown whether there is a small window of benefit in the short term, immediately after the application of the treatment.

Therefore, the primary aim of this study was to investigate the effect of radial shockwave therapy versus sham across three sessions on the immediate change in movement evoked pain. The secondary aim was to investigate the immediate change in pressure pain thresholds locally and remotely (as a potential mechanism).

## Materials and methods

This study was a parallel group and superiority randomised controlled trial. This study was reported in accordance with the Consolidated Standards of Reporting Trials statement^14^ and tendinopathy reporting standards.^15^ The template for treatment description and replication was used to describe the treatments.^16^ This trial was embedded in a larger trial testing the medium-term effects (up to 12 weeks) of radial shockwave versus sham, combined with an exercise treatment.^12^ The trial was prospectively registered in the Australian New Zealand Clinical Trials Registry, ACTRN12620000035921. Ethical approval for the trial was obtained from the Monash University Human Ethics Committee (21015), and all participants provided informed consent.

### Participants

Participants were recruited through Facebook and Instagram posts and our network of clinical partners. Participants aged 18 years or older with insertion Achilles tendinopathy for 3 months or longer were included. Insertional Achilles tendinopathy was diagnosed based on the presence of (i) gradual onset of pain in the posterior calcaneum region and (ii) self-reporting of pain during or after walking or running activities. Ultrasound imaging was used to confirm the presence of pathology. People were excluded if they had previously had shockwave treatment, Achilles tendon surgery, rupture (on the most painful side if pain was bilateral) or any other ankle conditions. People who self-reported having inflammatory conditions, neurological disorders or inherited connective tissue disorders were also excluded. Additionally, we excluded people who had used fluoroquinolone antibiotics within the past 2 years and anyone with an unstable mental health condition or other medical/social reasons that could impact on safety or adherence with the treatments. This trial was based at a single primary care private physiotherapy clinic in Melbourne, Australia.

### Interventions

Three physiotherapists were trained and all had prior experience in delivering radial shockwave to Achilles tendinopathy patients within their clinical practice (1–15 years of experience with radial shockwave). Participants received three sessions of radial shockwave, which included the baseline session and two subsequent sessions approximately 1 week apart (minimum of 5 and maximum of 10 days). During the treatments, participants were in a prone position. For those with bilateral insertional Achilles tendinopathy, the most affected side was chosen as the site of application. The most painful regions in the posterior calcaneum were marked for treatment (an area of about 2–3 cm^2^). The same education regarding radial shockwave and responses to questions about pain were delivered by physiotherapists at every session. The protocol for each trial arm (i.e. radial shockwave and sham) is described below.

*Radial shockwave:* Participants received 3000 shocks or pressure waves at a frequency of 10 Hz using a Chattanooga Intelect^®^ RPW GENERATION 2020 (Tennessee, USA) radial shockwave therapy device. The D-ACTOR^®^ applicator with a tip DI15, 15 mm, was used (estimated energy flux density [EFD] of 0.63 mJ/mm^2^ at a depth of 2–3 cm).^17^ Gel was applied to the skin to maximise conduction of pressure waves. The target was at least 5 out of 10 pain on a numerical rating scale (10 being the worst pain imaginable) during the entire session. Unless the participant was uncomfortable, then the aim was the most tolerable pain. The starting pressure was always two bars, and this was adjusted as needed (in 1 bar increments) during the session up to a maximum of 5 bars.

*Sham radial shockwave:* The procedure for sham radial shockwave was identical. The machine appeared and sounded the same, the only difference was that the applicator did not produce any pressure.

*Exercises and education:* Identical advice on exercise and education were delivered to both groups (Supplemental Files 1–3). The exercise was based on evidence published in the current literature^18,19^ and involved 4 sets of 15 repetitions of calf raises with knees bent (30 degrees of knee flexion) and knees straight performed 3 times (every second day) a week on flat ground with a 2-minute rest between sets and 1 minute between exercises. Progression of exercise was tailored to individual goals.

### Outcome measures

Movement evoked pain and pressure pain threshold outcomes were recorded pre and post each radial shockwave or sham session. Movement evoked pain was the primary outcome and was assessed with a standardised loading protocol immediately before and after each session. Participants self-reported their pain on a 100 mm visual analogue scale (zero = no pain; 100 = worst pain possible).^20^ The protocol included three single leg calf raises for people who did not undertake running sports and five single leg hops for people who did undertake running sports.

Pressure pain threshold was assessed at the area of radial shockwave application (the most affected insertional Achilles tendon), unaffected insertional Achilles tendon and the opposite elbow.^21^ A pressure algometer, FDX^®^ (Wagner FDX, Wagner Instruments, USA), was used to measure pressure pain threshold in kg/cm^2^ using reliable protocols.^22^

There were an additional two measures which helped to describe the radial shockwave and sham trial arms. First, the worst pain experienced during each radial shockwave or sham session was recorded using a 11-point numerical rating scale with terminal descriptors of 0 = no pain and 10 = worst pain imaginable. Second, the maximum bar level that was achieved during each session for both groups was also recorded.

### Sample size

The trial was powered to detect a minimal clinically important difference of 1.5 points on a 0-to-10-point numerical response scale for the primary outcome movement evoked pain.^23–26^ The assumed standard deviation for movement evoked pain was 2.1.^27^ G-Power 3.1 used to calculate a required sample size for an f^2^ effect size of 0.13 when specifying three tested predictors, controlling for up to 7 covariates. This sample size was adjusted for the three repeated sessions (three repeats of each outcome measure for each participant) utilising the design effect approach.^28^ A required sample size of 36 people per group (72 total) was calculated given a *p*-value of 0.05 and 80% power. We aimed for a recruitment target of 76 to account for potential attrition of 5%.

### Randomisation and blinding

Participants were randomised using permuted blocks (computer-generated permuted blocks of 4, 6 or 8) to either the radial shockwave or sham group by a researcher who had no contact with the participants (PM). Another researcher (not part of the author team) managed the randomisation from a distance at the clinic location by texting the physiotherapists administering the treatments to let them know to which group they were assigned. A single physiotherapist (BA) assessed outcomes and was blinded to treatment allocation. Only the two physiotherapists (not part of the author team) delivering the radial shockwave treatment were aware of treatment allocation (they followed a script [described below] to minimise bias). Blinding success was measured immediately following the initial radial shockwave or sham session by asking participants which group they believed they were assigned to (radial shockwave, sham, not sure). Procedures to ensure participant blinding included: (i) using identical treatment protocols and (ii) using the same machine that has the same appearance and emits the same noise for radial shockwave and sham (no pressure wave).

### Statistical analysis

IBM SPSS Statistics version 29 was used for all analyses. All randomised participants were included in the analysis (intention-to-treat).^29^ A linear mixed modelling approach was utilised to account for the repeated measures (random intercept) and the multivariate regression. Both outcomes (movement evoked pain and pressure pain threshold) were separately explored for their relationship with the following predictors: (1) group (radial shockwave or sham); (2) time (pre- or post-session) and (3) session (first, second or third); (4) along with all two-way and the three-way interaction of group × time × session. All analyses controlled for age, sex and body mass index. Our first step was to explore whether the three-way interaction was significant. In the absence of a significant three-way interaction term, we planned to remove the three-way interaction term and focus on evaluating the two-way interaction terms. The two-way interactions of interest included group × time (whether group has an influence on pre to post scores regardless of session) and time × session (helpful to explain whether any observed pre to post effect [regardless of group] is occurring only at certain sessions). The group × sessions interaction was not of interest as did not concern the pre to post effect of radial shockwave which was the focus of this study. Where interaction terms were significant, post hoc comparisons were conducted, to show significant mean differences in the outcomes across factors. A Bonferroni correction was used to control for multiple comparisons. Statistical tests for all predictors, and post hoc comparisons were two-tailed with a statistical significance set at 5%.

## Results

**Figure 1.**
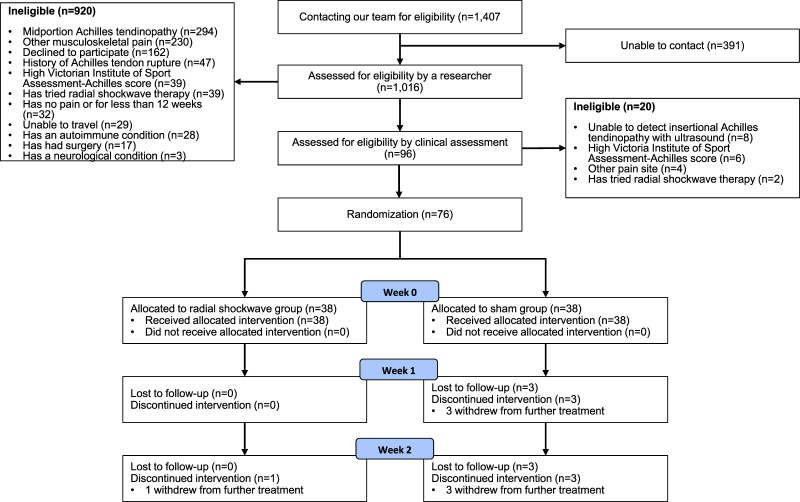
Flow diagram of follow up of participants in the radial shockwave and sham groups.

Figure 1 shows the flow of the participants through the trial. We screened 1407 participants for eligibility between April 2021 and November 2022. Seventy-six participants were recruited and randomised (40 female and 36 men and mean age 51 years). Thirty-eight were allocated to each group. Participants were comparable between groups for baseline characteristics (Table 1). Overall, 76, 73 and 73 participants completed the 0-, 1- and 2-week follow-up assessments, respectively (Figure 1).

**Table 1.** Participant characteristics.

	Radial shockwave	Sham
Age (year)	48.5 (12.2)	52.5 (11.1)
Sex female/male (%)	19/19 (50%)	21/17 (55%)
Weight (kg)	88 (24.7)	94.5 (18.7)
Height (cm^2^)	172.5 (10.1)	172.3 (10.3)
Body mass index (kg/m^2^)	29.4 (5.9)	31.9 (4.8)
Symptom duration (months)	33.3 (44.1)	47.8 (71)
Victoria Institute of Sports Assessment-Achilles	46.3 (13.6)	47.7 (16.2)
Pressure pain threshold at affected Achilles tendon	6.4 (2.8)	6.3 (3.0)
Pressure pain threshold at unaffected Achilles tendon	12.8 (4.2)	13.0 (4.4)
Pressure pain threshold at lateral elbow	10.3 (3.9)	9.4 (3.1)
Movement evoked pain	2.7 (1.7)	2.4 (1.7)

Values are mean (SD).

There were three participants in the sham group who did not attend the second and third sessions and one participant in the radial shockwave who did not attend the third session. Maximal pain during the 3 sessions was on average 7.3 (SD = 1.3) in the radial shockwave group and 3.2 (SD = 2.0) in the sham group (Table 2). The median bar pressure ranged from 1 to 1.7 in the radial shockwave across the 3 sessions (Table 2).

**Table 2.** Pain and treatment pressure during sessions.

	Pain during the treatment		*p-*Value
	Mean (95% CI)		
	Radial shockwave	Sham	
First treatment	7.6 (7.0 to 8.3)	3.8 (3.2 to 4.4)	<0.01
Second treatment	7.3 (6.8 to 7.9)	3.1 (2.5 to 3.6)	0.03
Third treatment	7.0 (6.4 to 7.6)	2.8 (2.2 to 3.4)	0.03

	Pressure		
	Median (IQR)		
First treatment	1.0 (0.7 to 1.8)	5.0 (5.0 to 5.0)	-
Second treatment	1.3 (1 to 1.3)	5.0 (5.0 to 5.0)	-
Third treatment	1.7 (1.8 to 2.6)	5.0 (5.0 to 5.0)	-

CI: confidence interval; IQR: interquartile range.

At the end of the first, second and third session, a comparable proportion of people in the sham (95%, 84% and 92%, respectively) and intervention group (100%, 95% and 100%, respectively) thought they were receiving the intervention or were not sure (Supplemental File 4).

### Primary and secondary outcome measures

Table 3 shows the results for the primary and secondary outcomes (adjusted analysis were not different so only the unadjusted findings are reported). The three-way interaction was not significant (F (2) = 1.218, p = 0.297). This means there was no evidence of a between group difference in a pre-to-post movement evoked pain scores across any of the three sessions. There was also no evidence of a between group difference in pre-to-post pressure pain threshold at the Achilles affected side (F (2) = 0.471, p = 0.625), Achilles unaffected side (F (2) = 0.503, p = 0.605) or elbow site (F (2) = 0.228, p = 0.796) after any of the sessions.

**Table 3.** Mean (SD) of movement evoked pain and pressure pain threshold at affected, unaffected Achilles and lateral elbow.

Outcome	Treatment	Radial shockwave		Sham	
		Mean (SD)		Mean (SD)	
		Pre	Post	Pre	Post
Movement evoked pain	1	4.3 (1.6)	3.1 (1.6)	4.4 (1.6)	2.8 (1.6)
	2	2.9 (1.6)	2.6 (1.6)	2.6 (1.8)	1.9 (1.8)
	3	2.4 (1.7)	2.1 (1.7)	1.6 (1.8)	1.7 (1.8)
Pressure pain threshold at affected Achilles	1	6.1 (3.1)	8.7 (3.1)	6.3 (3.1)	8.1 (3.1)
	2	6.1 (3.1)	8.0 (3.1)	6.6 (3.2)	8.7 (3.2)
	3	8.1 (3.1)	10.1 (3.1)	6.7 (3.2)	9.0 (3.2)
Pressure pain threshold at unaffected Achilles	1	12.4 (4.3)	13.8 (4.3)	12.3 (4.2)	13.2 (4.2)
	2	10.7 (4.3)	12.4 (4.3)	12.1 (4.3)	12.8 (4.3)
	3	11.0 (4.3)	12.1 (4.3)	11.4 (4.3)	12.9 (4.3)
Pressure pain threshold at lateral elbow	1	10.4 (3.4)	10.9 (3.4)	9.1 (3.4)	9.4 (3.4)
	2	9.6 (3.4)	10.7 (3.4)	9.2 (3.4)	9.9 (3.4)
	3	10.2 (3.4)	10.8 (3.4)	9.4 (3.4)	10.2 (3.4)

Movement evoked pain was measured on a 100 mm visual analogue scale (zero = no pain; 100 = worst pain possible). Pressure pain threshold was measured using kg/cm^2^.

**Figure 2.**
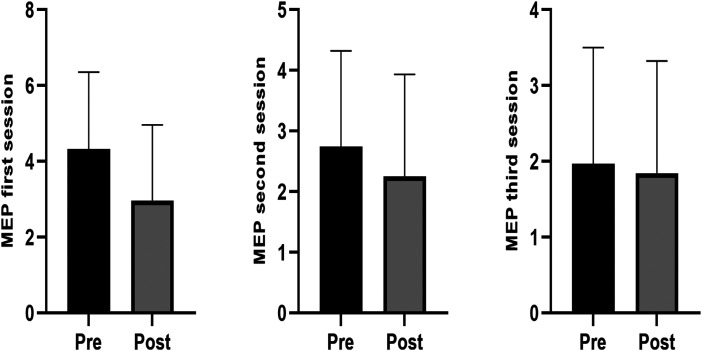
Changes in the movement evoked pain (MEP) scores pre to post after the three treatment sessions. MEP was measured on a 100 mm visual analogue scale (zero = no pain; 100 = worst pain possible). There were significant pre to post differences observed after the first (*p* = <0.01) and second (*p* = 0.03), but not the third (*p* = 0.57) treatment session in participants in both groups.

**Figure 3.**
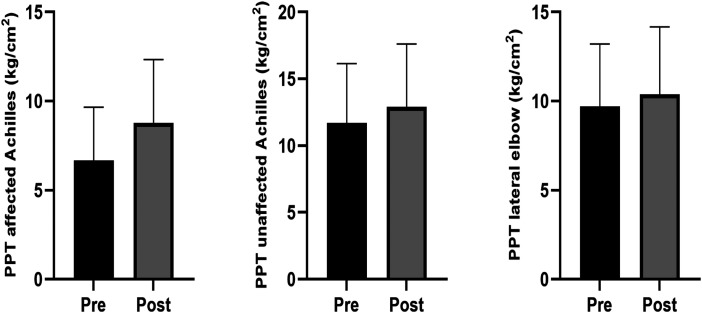
Changes in overall pressure pain threshold (PPT) testing pre- to post-treatment sessions. The average changes in pressure pain threshold over the three treatment sessions, measured using kg/cm^2^. There were significant pre to post differences observed in the pressure pain threshold testing for the affected (*p* = <0.01) and unaffected (*p* = <0.01) Achilles tendon, and lateral elbow (*p* = <0.01) in participants in both groups.

Excluding the three-way interaction we now explored the two-way interactions. For movement evoked pain, there was no group by time interaction (F (1) = 0.237, p = 0.627) but there was a session by time interaction (F (2) = 8.416, p ≤0.001). There was a small and significant pre-to-post reduction in movement evoked pain at the first (−1.4 [95% CI = −1.8 to −0.9]) and second sessions (−0.5 [95% CI = −0.9 to −0.1] (Figure 2). The change was trivial and non-significant at the third session (−0.1 [95% CI = −0.6 to 0.3]). For pressure pain threshold, there were no group by time interactions (Achilles affected: [F (1) = 0.071, p = 0.790]; Achilles unaffected: [F (1) = 0.473, p = 0.492]; elbow site: [F (1) = 0.285, p = 0.594]) or session by time interaction (Achilles affected: F (2) = 0.063, p = 0.939; Achilles unaffected: [F (1) = 0.052, p = 0.949]; elbow site: [F (1) = 0.801, p = 0.450]) at any site, so main effects are reported. There were small and significant pre-to-post increases in pressure pain threshold at each site regardless of group or week (Achilles affected side: 2.1 [95% CI = 1.7 to 2.6]; Achilles unaffected side: 1.2 [0.7 to 1.8]; elbow site: 0.7 [95% CI = 0.3 to 1.1]), as shown in Figure 3. Unadjusted data are show as adjusted as they were very similar. For all analyses, adjusted analysis were not different so only the unadjusted data are reported.

## Discussion

This randomised trial found radial shockwave therapy to be no more efficacious than a sham for immediately reducing movement evoked pain in people with insertional Achilles tendinopathy. This is supported by the findings of no between-group difference in the movement evoked pain or pressure pain threshold outcomes after any of the three sessions. There was a small and significant reduction in movement evoked pain in both groups (average 1.1 on a 0-to-10-point numerical rating scale), which approximated, but did not exceed the minimum important difference of 1.5 points. Further, this improvement in movement evoked pain was only seen after the first session. There was also a small and significant increase in pressure pain threshold (kg/cm^2^) in both groups following all interventions (2.1 on affected Achilles insertion, 1.2 on unaffected, 0.7 on elbow on average). However, the immediate improvements observed in these outcomes were not influenced by group allocation and are most likely explained by non-specific effects, such as the expectation of recovery or familiarisation with the tests and testing environment.^30^

It is unclear why participants had reduced movement evoked pain after the first session but was not sustained in subsequent sessions. Plausible explanations could be contextual factors, in particular, the expectation of intervention success drove the movement evoked pain response that we observed. Expectation is likely to be heightened at the beginning of an intervention, especially as none of the participants in our trial had previously had radial shockwave. It is possible that if people did not experience the lasting pain benefits after the first session of radial shockwave that they may have expected, this reduced the efficacy of subsequent sessions.^31^ Alternatively, it is also possible that the exercise and education delivered reduced their pain over the first 3 weeks of the trial to such an extent that it may have impaired their ability to identify radial shockwave effects on movement evoked pain in the second and third sessions. This is supported by our findings that between sessions 1 and 3 there was a trend for the participants movement evoked pain to decrease, with an average starting point of 4.4 pain and ending at 1.9 points.

Immediate effects of radial shockwave have been investigated for other musculoskeletal conditions. Walewicz et al.^8^ investigated the immediate effects of radial shockwave on lower back pain in two groups (radial shockwave and sham) and found that the radial shockwave group experienced greater pain reduction immediately after intervention compared to the sham group. This contrasts with our findings, and it may be that there are different effects of radial shockwave with different conditions and pathologies. Alternatively, Walewicz et al.^8^ applied two interventions a week over 5 weeks, which may suggest that a greater dose is needed to observe an effect. This is the first trial to investigate the immediate effects of radial shockwave on pain and pressure pain threshold outcomes among people with insertional Achilles tendinopathy. The most recent clinical practice guidelines for insertional Achilles tendinopathy recommend radial shockwave when education and exercise are not proving effective.^32,33^ However, given that we found no between-group difference and radial shockwave is severely painful and distressing, it could be argued that this treatment should not be used as a short-term intervention for reducing pain in people with insertional Achilles tendinopathy.

Key strengths of this trial include its rigorous design, participant blinding, and adequate statistical power for the primary outcome. In addition, we also incorporated measures of pressure pain threshold to understand the mechanism of action of radial shockwave in people with insertional Achilles tendinopathy. However, our findings should be interpreted in context of several limitations. First, as is the case with all trials investigating mechanical intervention/devices, our control intervention cannot be considered truly inert and is best considered as a sham rather than a placebo. Our findings indicate there is no significant benefit from real radial shockwave compared to a device held to the skin, which could be replicated as simply as pressing against the Achilles tendon. The energy level delivered (10 Hz, 3000 shocks, 2–4 bar) is approximately 0.63 mJ/mm², but the minimum effective dose remains uncertain.^7^ Second, due to the nature of the interventions, the physiotherapist administering the radial shockwave could not be blinded to group allocation, though their interactions were scripted to reduce bias. Third, we did not measure the use of co-interventions, which the participants may have been using in addition to the interventions used in this trial and may have contributed to the improvements observed. Finally, our participants were volunteers recruited from the general community, primarily through social media and clinical networks, and may have excluded other potential participants who are digitally isolated or not well connected to our clinical networks.

In conclusion, radial shockwave therapy demonstrated no more efficacy than a sham in reducing immediate movement evoked pain in adults with insertional Achilles tendinopathy. In addition, there was no change in pressure pain threshold testing. Any benefit observed with this intervention is most likely driven by contextual factors, such as familiarisation or expectation of recovery. These findings do not support the use of radial shockwave therapy as a primary management approach for insertional Achilles tendinopathy.

## Clinical messages

- Radial shockwave therapy is no more efficacious than a sham in reducing immediate movement evoked pain in adults with insertional Achilles tendinopathy.
- Any observed benefit with the use of radial shockwave is likely driven by non-specific effects, rather than the currently held belief of conditioned pain modulation.

## Supplemental Material

sj-docx-1-cre-10.1177_02692155251394951 - Supplemental material for Does radial shockwave therapy lead to immediate improvements in pain in people with insertional Achilles tendinopathy? A randomised controlled trial Supplemental material, sj-docx-1-cre-10.1177_02692155251394951 for Does radial shockwave therapy lead to immediate improvements in pain in people with insertional Achilles tendinopathy? A randomised controlled trial by B Alsulaimani, L Perraton, J Bourke, T Powers and P Malliaras in Clinical Rehabilitation

sj-docx-2-cre-10.1177_02692155251394951 - Supplemental material for Does radial shockwave therapy lead to immediate improvements in pain in people with insertional Achilles tendinopathy? A randomised controlled trial Supplemental material, sj-docx-2-cre-10.1177_02692155251394951 for Does radial shockwave therapy lead to immediate improvements in pain in people with insertional Achilles tendinopathy? A randomised controlled trial by B Alsulaimani, L Perraton, J Bourke, T Powers and P Malliaras in Clinical Rehabilitation

sj-docx-3-cre-10.1177_02692155251394951 - Supplemental material for Does radial shockwave therapy lead to immediate improvements in pain in people with insertional Achilles tendinopathy? A randomised controlled trial Supplemental material, sj-docx-3-cre-10.1177_02692155251394951 for Does radial shockwave therapy lead to immediate improvements in pain in people with insertional Achilles tendinopathy? A randomised controlled trial by B Alsulaimani, L Perraton, J Bourke, T Powers and P Malliaras in Clinical Rehabilitation

sj-docx-4-cre-10.1177_02692155251394951 - Supplemental material for Does radial shockwave therapy lead to immediate improvements in pain in people with insertional Achilles tendinopathy? A randomised controlled trial Supplemental material, sj-docx-4-cre-10.1177_02692155251394951 for Does radial shockwave therapy lead to immediate improvements in pain in people with insertional Achilles tendinopathy? A randomised controlled trial by B Alsulaimani, L Perraton, J Bourke, T Powers and P Malliaras in Clinical Rehabilitation

## References

[1] Chen J Janney CF Khalid MA , et al. Management of insertional Achilles tendinopathy. J Am Acad Orthop Surg 2022; 30: e751–e7e9. 10.5435/JAAOS-D-21-00679 35286285

[2] Gaston TE Daniel JN . Achilles insertional tendinopathy – is there a gold standard? Arch Bone Jt Surg 2021; 9: 5–8. 33778110 10.22038/abjs.2020.53988.2704 PMC7957111

[3] Jarin IJ Bäcker HC Vosseller JT . Functional outcomes of insertional Achilles tendinopathy treatment: a systematic review. JBJS Rev 2021; 9. DOI: 10.2106/JBJS.RVW.20.00110. 34125735

[4] Sayana MK Maffulli N . Eccentric calf muscle training in non-athletic patients with Achilles tendinopathy. J Sci Med Sport 2007; 10: 52–58. 16828343 10.1016/j.jsams.2006.05.008

[5] Simplicio CL Purita J Murrell W , et al. Extracorporeal shock wave therapy mechanisms in musculoskeletal regenerative medicine. J Clin Orthop Trauma 2020; 11: S309–S318. 10.1016/j.jcot.2020.02.004 PMC7275282 32523286

[6] Malliaras P . Physiotherapy management of Achilles tendinopathy. J Physiother 2022; 68: 221–237. 36274038 10.1016/j.jphys.2022.09.010

[7] Samhan AF Abdelhalim NM . Impacts of low-energy extracorporeal shockwave therapy on pain, pruritus, and health-related quality of life in patients with burn: a randomized placebo-controlled study. Burns 2019; 45: 1094–1101. 30827852 10.1016/j.burns.2019.02.007

[8] Walewicz K Taradaj J Dobrzyński M , et al. Effect of radial extracorporeal shock wave therapy on pain intensity, functional efficiency, and postural control parameters in patients with chronic low back pain: a randomized clinical trial. J Clin Med 2020; 9: 568. 32092987 10.3390/jcm9020568 PMC7074373

[9] Hausdorf J Lemmens MA Heck KD , et al. Selective loss of unmyelinated nerve fibers after extracorporeal shockwave application to the musculoskeletal system. Neuroscience 2008; 155: 138–144. 18579315 10.1016/j.neuroscience.2008.03.062

[10] Vallance P Malliaras P . Extracorporeal shockwave therapy of healthy Achilles tendons results in a conditioned pain modulation effect: a randomised exploratory cross-over trial. Muscles Ligaments Tendons J 2019; 9: 262–271.

[11] Vallance P Crowley L Vicenzino B , et al. Contralateral mechanical hyperalgesia and altered pain modulation in men who have unilateral insertional Achilles tendinopathy: a cross-sectional study. Musculoskelet Sci Pract 2021; 52: 102353. 33636582 10.1016/j.msksp.2021.102353

[12] Alsulaimani B Perraton L Vallance P , et al. Does shockwave therapy lead to better pain and function than sham over 12 weeks in people with insertional Achilles tendinopathy? A randomised controlled trial. Clin Rehabil 2025; 39: 174–186. 39704142 10.1177/02692155241295683 PMC11846266

[13] Mansur NSB Matsunaga FT Carrazzone OL , et al. Shockwave therapy plus eccentric exercises versus isolated eccentric exercises for Achilles insertional tendinopathy: a double-blinded randomized clinical trial. J Bone Joint Surg Am 2021; 103: 1295–1302. 34029235 10.2106/JBJS.20.01826

[14] Schulz KF Altman DG Moher D , et al. CONSORT 2010 statement: updated guidelines for reporting parallel group randomised trials. Int J Surg 2011; 9: 672–677. 22019563 10.1016/j.ijsu.2011.09.004

[15] Rio EK Mc Auliffe S Kuipers I , et al. ICON PART-T 2019-International Scientific Tendinopathy Symposium Consensus: recommended standards for reporting participant characteristics in tendinopathy research (PART-T). Br J Sports Med 2020; 54: 627–630. 31519545 10.1136/bjsports-2019-100957

[16] Hoffmann TC Glasziou PP Boutron I , et al. Better reporting of interventions: template for intervention description and replication (TIDieR) checklist and guide. Br Med J 2014; 348: g1687. 10.1136/bmj.g1687 24609605

[17] Chattanooga. Intelect RPW shockwave brochure, https://enovis-medtech.eu/media/storage.djoglobal.eu/en_US/Documents/Support_documents/IFU_13-28670_US_Rev_A_Intelect_RPW_2_DIGITAL_Final.pdf.

[18] Malliaras P Barton CJ Reeves ND , et al. Achilles and patellar tendinopathy loading programmes: a systematic review comparing clinical outcomes and identifying potential mechanisms for effectiveness. Sports Med 2013; 43: 267–286. 23494258 10.1007/s40279-013-0019-z

[19] Murphy MH Lahart I Carlin A , et al. The effects of continuous compared to accumulated exercise on health: a meta-analytic review. Sports Med 2019; 49: 1585–1607. 31267483 10.1007/s40279-019-01145-2 PMC6745307

[20] Cook JL Purdam CR . Is tendon pathology a continuum? A pathology model to explain the clinical presentation of load-induced tendinopathy. Br J Sports Med 2009; 43: 409–416. 18812414 10.1136/bjsm.2008.051193

[21] Arendt-Nielsen L Yarnitsky D . Experimental and clinical applications of quantitative sensory testing applied to skin, muscles and viscera. J Pain 2009; 10: 556–572. 19380256 10.1016/j.jpain.2009.02.002

[22] Park G Kim CW Park SB , et al. Reliability and usefulness of the pressure pain threshold measurement in patients with myofascial pain. Ann Rehabil Med 2011; 35: 412–417. 22506152 10.5535/arm.2011.35.3.412 PMC3309218

[23] Ozgüçlü E Cetin A Cetin M , et al. Additional effect of pulsed electromagnetic field therapy on knee osteoarthritis treatment: a randomized, placebo-controlled study. Clin Rheumatol 2010; 29: 927–931. 20473540 10.1007/s10067-010-1453-z

[24] Pinitkwamdee S Laohajaroensombat S Orapin J , et al. Effectiveness of extracorporeal shockwave therapy in the treatment of chronic insertional Achilles tendinopathy. Foot Ankle Int 2020; 41: 403–410. 31924120 10.1177/1071100719898461

[25] Schroeder AN Tenforde AS Jelsing EJ . Extracorporeal shockwave therapy in the management of sports medicine injuries. Curr Sports Med Rep 2021; 20: 298–305. 34099607 10.1249/JSR.0000000000000851

[26] Tashjian RZ Deloach J Porucznik CA , et al. Minimal clinically important differences (MCID) and patient acceptable symptomatic state (PASS) for visual analog scales (VAS) measuring pain in patients treated for rotator cuff disease. J Shoulder Elbow Surg 2009; 18: 927–932. 19535272 10.1016/j.jse.2009.03.021

[27] Chimenti RL Post AA Rio EK , et al. The effects of pain science education plus exercise on pain and function in chronic Achilles tendinopathy: a blinded, placebo-controlled, explanatory, randomized trial. Pain 2023; 164: e47–e65. 10.1097/j.pain.0000000000002720 PMC10016230 36095045

[28] Snijders TA . Power and sample size in multilevel modeling. Ency Stat Behav Sci 2005; 3: 1573.

[29] Klyne DM Moseley GL Sterling M , et al. Individual variation in pain sensitivity and conditioned pain modulation in acute low back pain: effect of stimulus type, sleep, and psychological and lifestyle factors. J Pain 2018; 19: 942.e1–942.e18. 10.1016/j.jpain.2018.02.017 29597080

[30] Cook CE Bailliard A Bent JA , et al. An international consensus definition for contextual factors: findings from a nominal group technique. Front Psychol 2023; 14: 1178560. 37465492 10.3389/fpsyg.2023.1178560 PMC10351924

[31] Colloca L . The placebo effect in pain therapies. Annu Rev Pharmacol Toxicol 2019; 59: 191–211. 30216744 10.1146/annurev-pharmtox-010818-021542 PMC6402571

[32] Chimenti RL Cychosz CC Hall MM , et al. Current concepts review update: insertional Achilles tendinopathy. Foot Ankle Int 2017; 38: 1160–1169. 28789557 10.1177/1071100717723127 PMC5956523

[33] de Vos RJ van der Vlist AC Zwerver J , et al. Dutch Multidisciplinary guideline on Achilles tendinopathy. Br J Sports Med 2021; 55: 1125–1134. 34187784 10.1136/bjsports-2020-103867 PMC8479731

