# Supplementary material for: Does radial shockwave therapy lead to immediate improvements in pain in people with insertional Achilles tendinopathy? A randomised controlled trial
Source: Clin Rehabil. 2025 Nov 27;40(2):171–81. doi: 10.1177/02692155251394951 (PMC12816402; doi:10.1177/02692155251394951)
Supplement: sj-docx-1-cre-10.1177_02692155251394951 - Supplemental material for Does radial shockwave therapy lead to immediate improvements in pain in people with insertional Achilles tendinopathy? A randomised controlled trial [file sj-docx-1-cre-10.1177_02692155251394951.docx]

**Supplementary File 1:** Education sheet

Achilles Tendinopathy General Information

- Achilles tendon pain is common
- Change in activities that load the Achilles tendon is the most common cause
- Advice and exercise are the most important treatments, but some people require other treatments like shockwave therapy.

**The Achilles tendon**

| A tendon attaches muscles to bone. Your Achilles tendon is found at the back of the lower leg, just above the heel bone. It attaches your two calf muscles (gastrocnemius and soleus) to the heel bone (calcaneus) and helps you go up onto tiptoes.  The Achilles tendon is the biggest and strongest tendon in the body. Its main role is to transfer load from muscle to bone. | 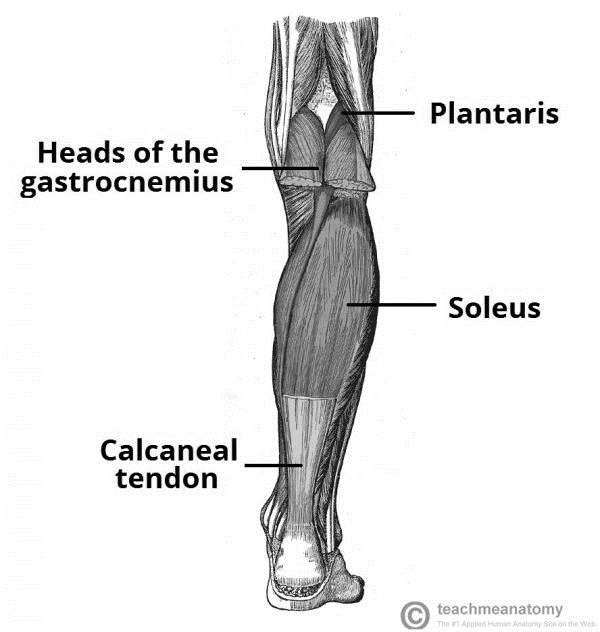 |
| --- | --- |

**What is Achilles tendinopathy?**

This condition is characterised by pain in the Achilles tendon, previously referred to as Achilles tendinitis. There are two types of Achilles tendinopathy: Mid-portion, where pain is usually present in the middle portion of the Achilles tendon; and insertional, which affects the spot where the tendon meets the heel bone. There are many risk factors for Achilles tendinopathy, including:

- **Changes in activity** – this occurs when new activities surpass the capacity of the tendon. For example, when starting to walk or run more, or starting to run up hills more. Athletes can develop Achilles tendinopathy, but it is also a common overuse condition in people not involved in sport.
- **Older age**
- **Metabolic issues** e.g. elevated cholesterol or diabetes.
- **Overweight and obesity** – being overweight places more strain on the Achilles tendon.
- **Onset of menopause** in females

**Common symptoms of Achilles tendinopathy**

- **Morning stiffness:** Many people complain of stiffness around the tendon when they get up in the morning. This usually eases after a few minutes of walking, but may last longer.
- **Tenderness over the Achilles tendon:** Often the tendon is very tender to touch when gently squeezed. There may be a tender lump in the tendon.
- **Pain with exercise:** Pain during or after weight-bearing activity (such as walking, running and jumping) is common. For some people, the pain improves with activity. Others experience severe pain in their Achilles tendon which stops them from doing their activity.

**How is Achilles tendinopathy diagnosed?**

It can be diagnosed by health care professionals who treat Achilles tendon pain such as a sports doctor, general practitioner, physiotherapist or podiatrist. Diagnosis is based on asking questions to identify the potential cause and factors that aggravate your pain, for example, type and frequency of exercises etc. A physical examination will be required as well as testing your movements and leg strength.

**Do I need a scan?**

We don’t always need to carry out X-rays or scans (imaging) to diagnose Achilles tendinopathy. However, an ultrasound scan may sometimes be needed if the diagnosis is not clear. Ultrasound is a quick, safe and effective way for us to see your tendon. Magnetic Resonance Imaging (MRI) may also be used, but this is not common.

**What are the treatments for Achilles tendinopathy?**

***The first aim of the treatment*** is to reduce pain. This can be achieved by:

- Avoiding or limiting activities that may aggravate the condition, such as excessive walking and running. This can be replaced with activities that do risk aggravating Achilles pain such as swimming or cycling.
- Applying ice to the tendon
- Gentle exercises

***The second aim of treatment*** is to perform specific exercises that strengthen the tendon and associated muscles and this may also help your pain.

***The third aim of treatment*** is to gradual resume activities that you have had to reduce due to your pain. This involves starting with a few minutes and increasing the time spent doing these activities over a number of weeks. Monitor your pain during and after exercises. Do the activity with less than 5/10 pain. If you experience more pain, then reduce the activity by 50% or stop completely, and resume when the pain is improved. We will guide you during this process.

**What about medications?**

If you feel you really need more pain cover, you could take paracetamol 500mg. See you doctor if you are not sure whether you are able to take this.

**Prognosis**

Since the tendon weakening (degeneration) usually occurs over months or years prior to onset of symptoms; recovery may take a few months. Most people experience significant improvement in their Achilles tendon pain and ability to perform their normal activities with the right advice and exercise over **a period of 12 weeks**.

**Why is exercise recommended if it was the cause of my Achilles problem?**

People with Achilles tendinopathy sometimes find it strange that exercise is recommended to treat Achilles tendon pain when too much activity often causes it. However, introducing appropriate exercises gradually is a very effective way to increase the capacity of your Achilles tendon by making the muscles and tendon stronger and more resistant to loading. Please refer to the exercise booklet for further details on the type of exercises and how to progress gradually.

**But it hurts! Is it normal to feel pain during activity and exercise for my Achilles tendon pain?**

Some people avoid exercises completely out of fear they will cause further damage. It is common and in fact quite normal to feel some pain when performing or after performing exercises. This pain usually settles within a reasonable period of time, less than a day. Some pain with exercise is NOT a sign of damage.


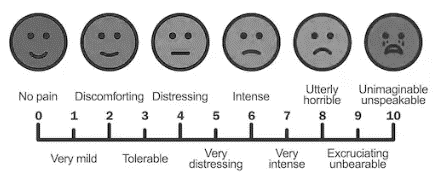
If you feel an activity or exercise is too painful, stop doing the exercise or modify how you do it.

‘Too painful’ is defined as equal to or more than 5/10 pain. When your pain settles again, it is safe and suggested to recommence the activity or the exercise.

**Other treatments like shockwave and surgery**

Treatments like shockwave are usually considered if there’s no change in symptoms after trying the above suggested treatments. Other options like injection may also be helpful. Your treating practitioner will discuss these options with you.

Surgery is the last resort if other treatments have failed. It is not guaranteed to relieve your symptoms.
