# Supplementary material for: Does radial shockwave therapy lead to immediate improvements in pain in people with insertional Achilles tendinopathy? A randomised controlled trial
Source: Clin Rehabil. 2025 Nov 27;40(2):171–81. doi: 10.1177/02692155251394951 (PMC12816402; doi:10.1177/02692155251394951)
Supplement: sj-docx-2-cre-10.1177_02692155251394951 - Supplemental material for Does radial shockwave therapy lead to immediate improvements in pain in people with insertional Achilles tendinopathy? A randomised controlled trial [file sj-docx-2-cre-10.1177_02692155251394951.docx]

**Supplementary File 2:** Exercise sheet

**EXERCISE INFORMATION**

As a participant of this research study you are expected to perform an exercise training program. There are some important guidelines to observe whilst performing the exercises:

You will need to perform the exercises on both legs alternatively as per the instructions below:

**TEMPO:**

3 seconds up, 3 seconds down

**FREQUENCY:**

3x/week

**REPETITIONS:** 15 repetitions x 4 sets (with 2 minutes rest between sets and 1 minute between Exercise A and Exercise B)

**It is very important to note that during this exercise program you may experience some pain and/or fatigue.** Continue the exercise even if you experience pain up to 5 out of 10, based on the pain scale below. The pain will reduce as you continue your rehabilitation.

**PAIN SCALE**


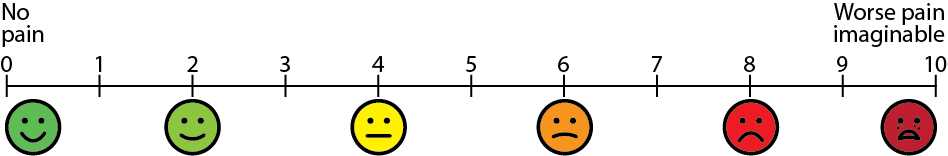


To help you keep track of these exercises and your symptoms, record your progress at the end of this document.

1.
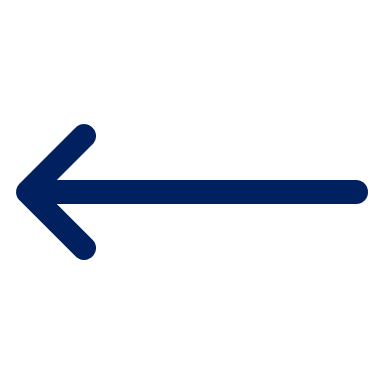
**Exercise A: Calf raise with straight knee**

**Knee straight: Level 2**

**Knee straight: Level 1**

Stand upright on a flat surface next to a wall. Use the wall to balance yourself if required.

Standing on one leg, lift the heel **as high as possible** on your weight bearing leg, rising up onto your toes.

Slowly lower the heel down again

Make sure you **keep the knee straight** throughout the exercise

**Level 2:** Add 5kg weight to increase the load on the tendon, either in one hand or back-pack (rice packets, books, bricks etc).

Continue increasing in 5kg


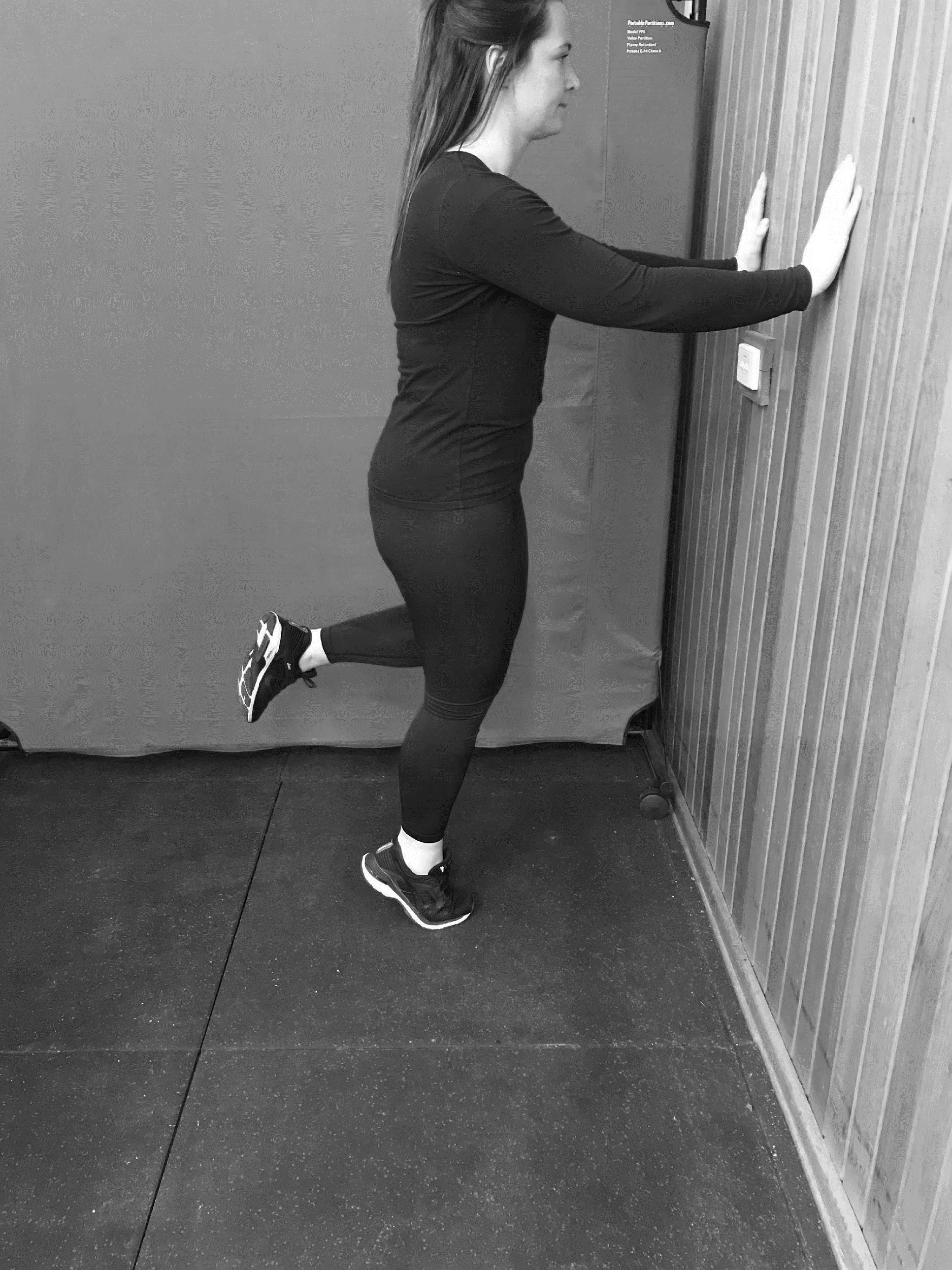

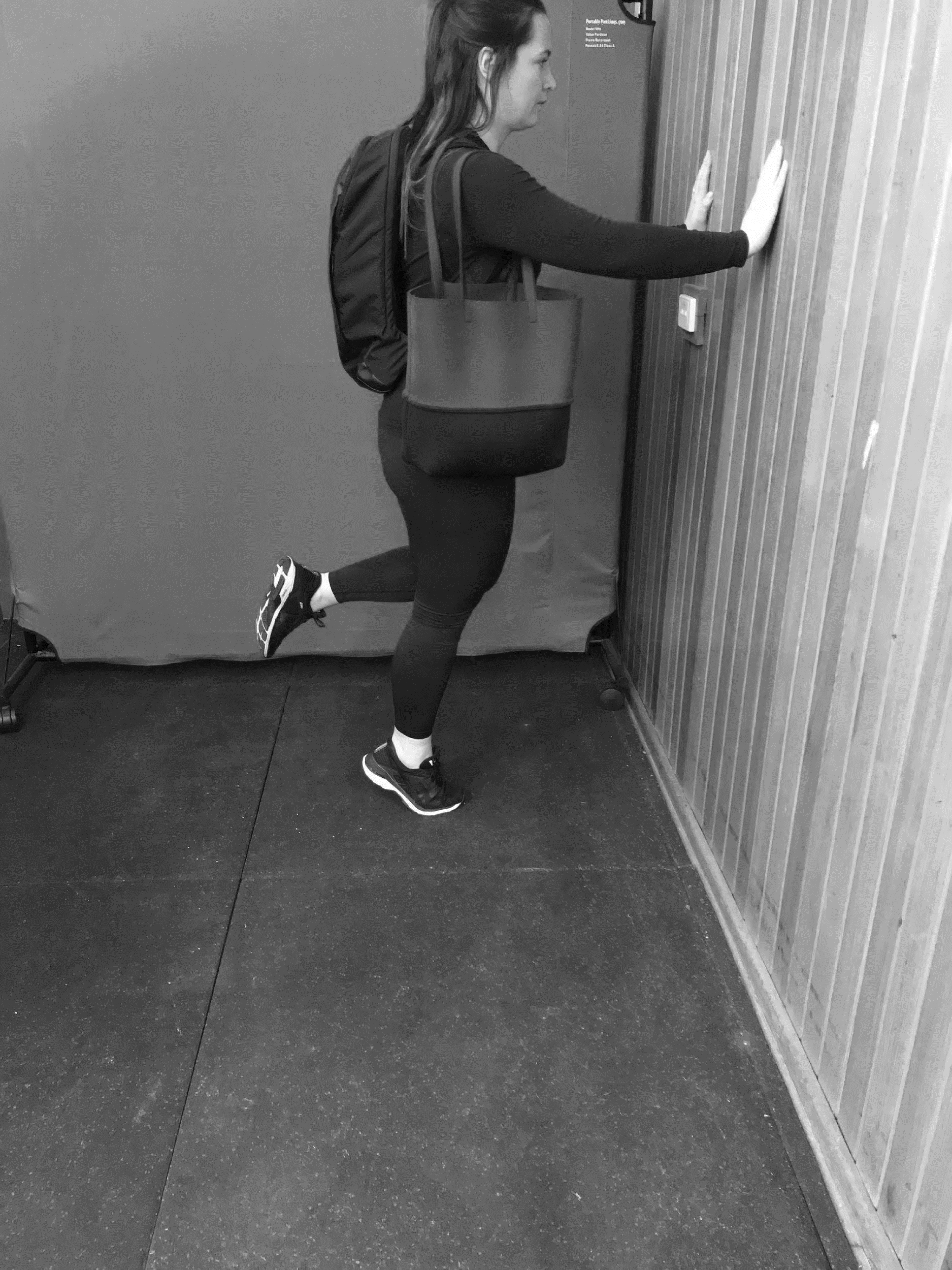


| **Is this exercise too easy or difficult?** |
| --- |
| Refer to the modifications table for this exercise and follow the steps to make it more suitable for you. |

1. **Exercise B: Calf raise with bent knee**

Stand leaning forward on a wall. Bend knee to approximately 20-30 degrees of bend.

Standing on one leg, lift the heel **as high as possible** on your weight bearing leg

Slowly lower the heel down again

Make sure you **keep the knee bent** throughout the entire movement

**Level 2:** Add 5kg weight to increase the load on the tendon, either in one hand or back-pack (rice packets, books, bricks etc). Continue increasing in 5kg increments as tolerated.

**Knee bent: Level 1**

**Knee bent: Level 2**


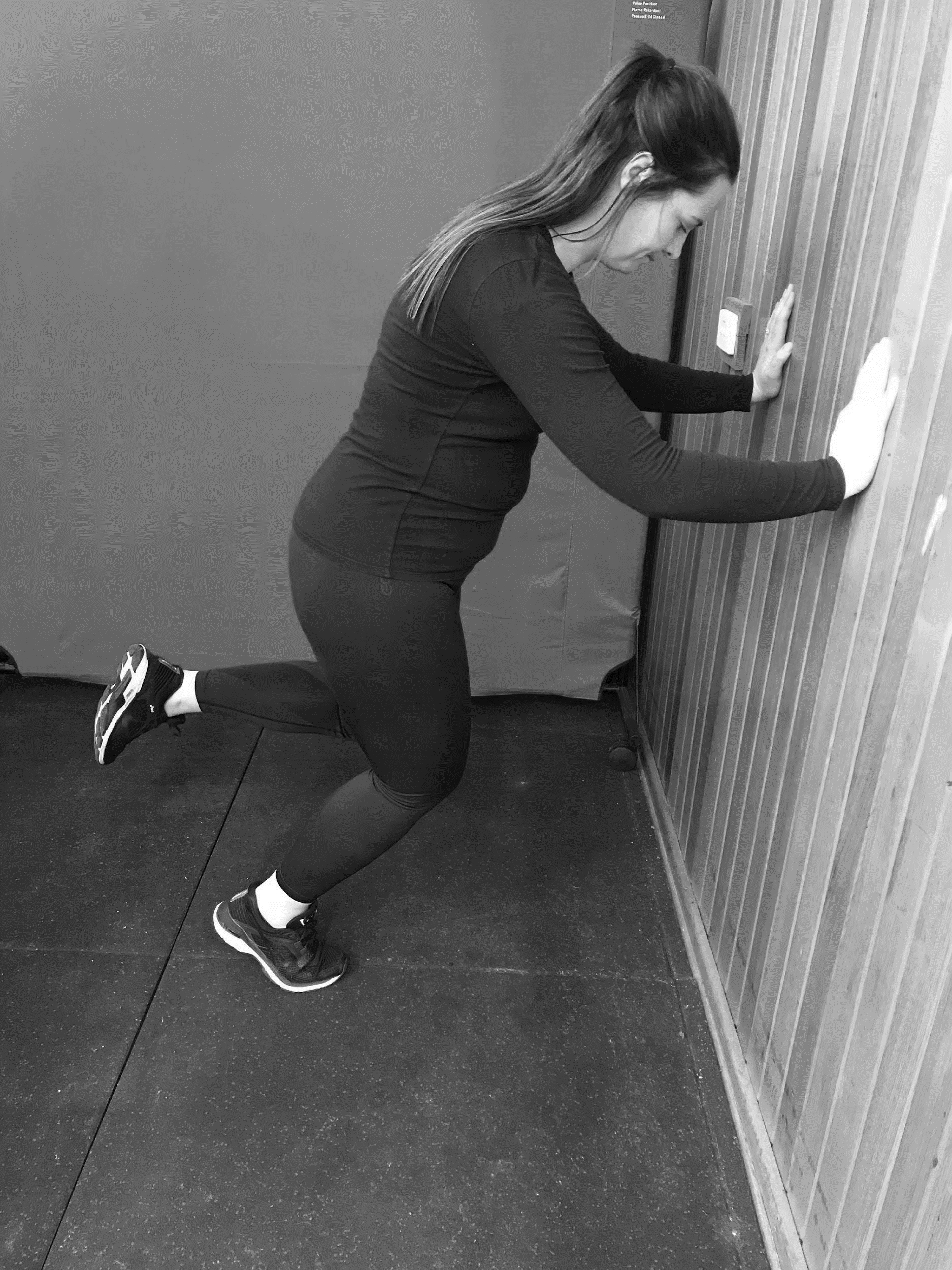

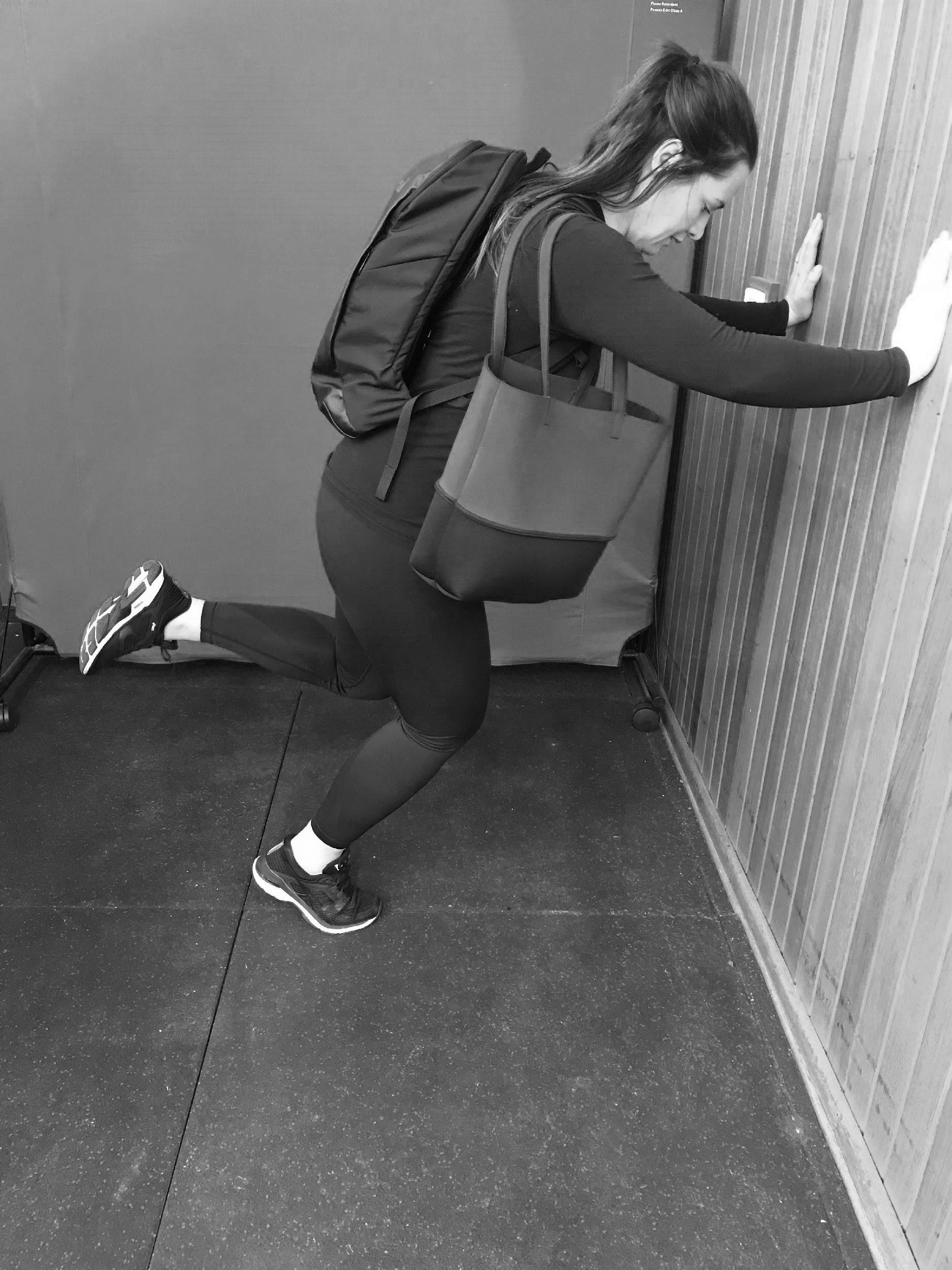


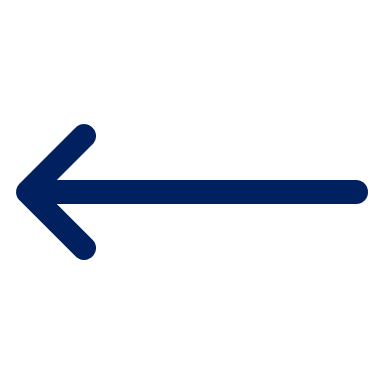


| **Is this exercise too easy or difficult?** |
| --- |
| Refer to the modifications table for this exercise and follow the steps to make it more suitable for you. |

**Modification table**


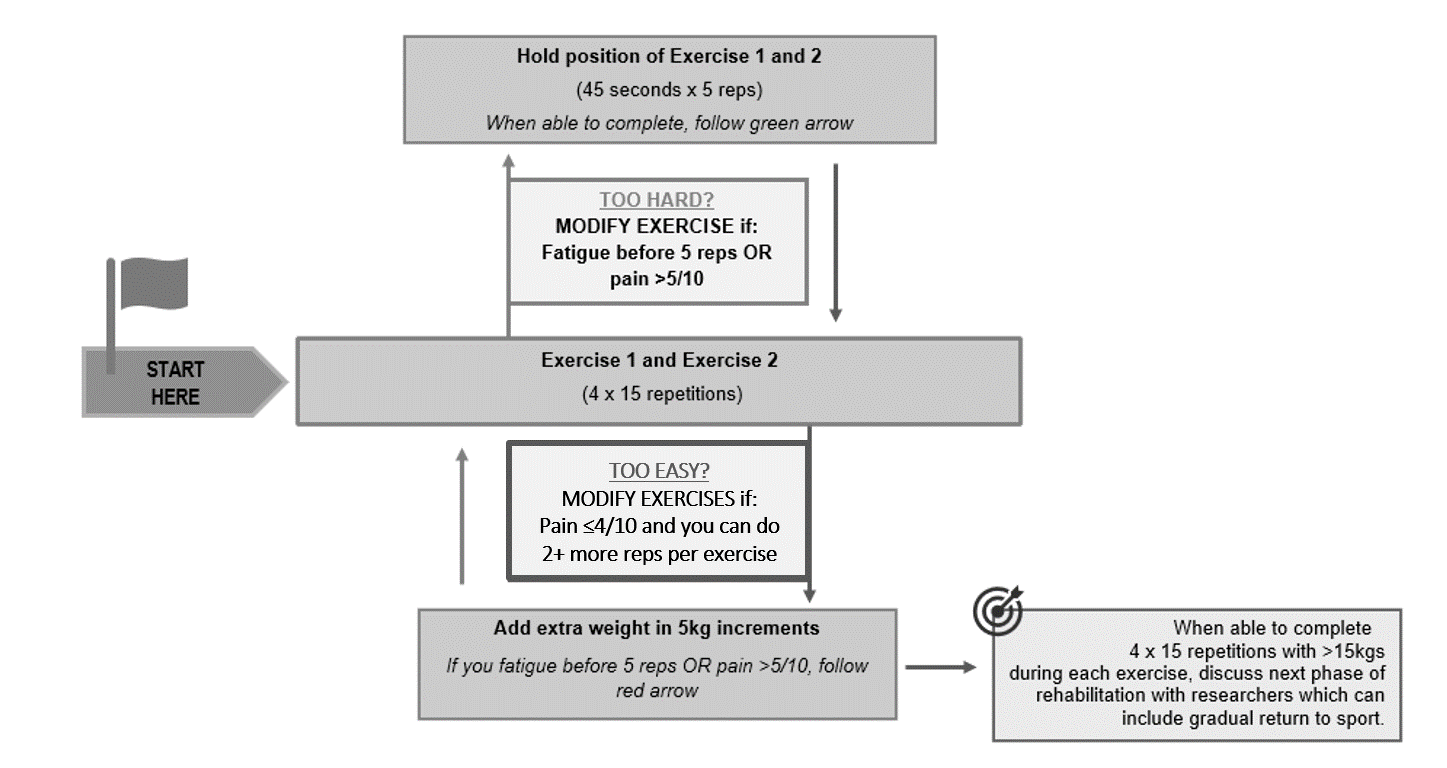


**Education and exercise diagram
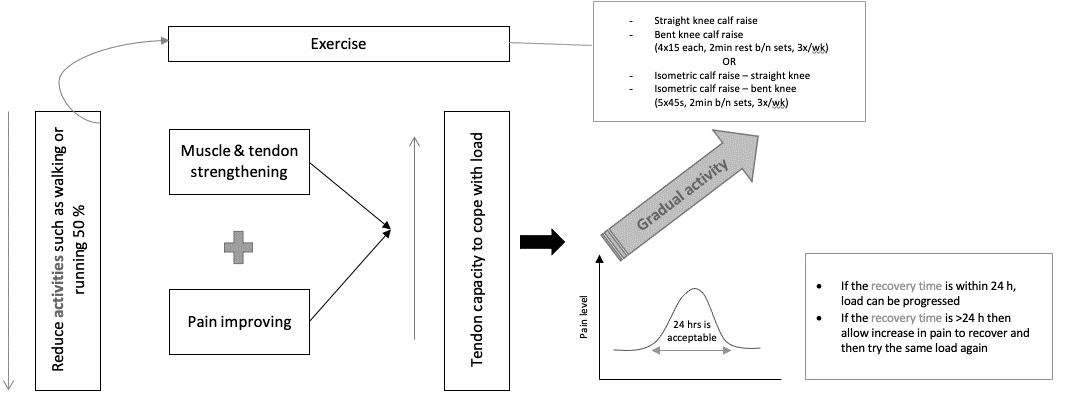
**

**How to progress and regress your exercise**

| **LOAD TEST (do 4-5 times)** | **Pain ‘acceptable’ = ≤4/10** | **Pain 5-7/10** | **Pain ≤8/10** |
| --- | --- | --- | --- |
| IF YOU ARE A WALKER:  5 x 1 leg calf raise | - Isotonic is ok - If you can do 4 or more reps at the end add 5kg - Add a maximum of 5kg per week - Your goal should be 10-15kg (walkers) or 20-25kg (runners) | - Regress to isometric - Keep adding 5kg per week if you are finding you can do it easily - Also reduce walking, running, running sports by 50% - Stop any intensity (e.g. hill or fast walking/running) | - Regress to isometric - Also reduce walking to only incidental and stop all running/running sports |
| IF YOU ARE A RUNNER:  5 x 1 leg calf raise  AND  5 x 1 leg continuous hops |  |  |  |
| Progressing walking/running | - Not for the first 2 weeks | - Test load tests every session to see if you are ready to progress | Test load tests every session to see if you are ready to progress |

**A guide to progressing walking/running**

|  | 10% | 20% |
| --- | --- | --- |
| Full time/ minutes | **Minutes** | **Minutes** |
| 10 | 1 | 2 |
| 20 | 2 | 4 |
| 30 | 3 | 6 |
| 30 | 3 | 6 |
| 40 | 4 | 8 |
| 60 | 6 | 12 |

**MY GOALS**

In this section please write down your weight goals. An example might be to be able to perform 4 sets of 15 of the calf raise exercise with 30kg by the end of the program (12 weeks).

**Exercise adherence**

In this section, please keep a record of the exercises you have done. For example:

| **Date**  **1** | **Exercises / Repetitions / Weight** | | | | | | |
| --- | --- | --- | --- | --- | --- | --- | --- |
|  | **Exercise 1 (with knee straight)** | Did you do the prescribed 4 sets of 15 repetitions? | Exercise weight (kg) | Step added | **Exercise 2 (with knee bent)** | Did you do the prescribed 4 sets of 15 repetitions? | Exercise weight (kg) |
| **Date**  **2** | Yes/No | Yes/No  If no, how many______  why? __________________________ |  | Yes/No | Yes/No | Yes/No  If no, how many______  Why? ______________________ |  |
| **Date**  3 | Yes/No | Yes/No  If no, how many______  why? __________________________ |  | Yes/No | Yes/No | Yes/No  If no, how many______  Why? ______________________ |  |
| **Date**  **4** | Yes/No | Yes/No  If no, how many______  why? __________________________ |  | Yes/No | Yes/No | Yes/No  If no, how many______  Why? ______________________ |  |
| **Date** | Yes/No | Yes/No  If no, how many______  why? __________________________ |  | Yes/No | Yes/No | Yes/No  If no, how many______  Why? ______________________ |  |
| **Date** | Yes/No | Yes/No  If no, how many______  why? __________________________ |  | Yes/No | Yes/No | Yes/No  If no, how many______  Why? ______________________ |  |
| **Date** | Yes/No | Yes/No  If no, how many______  why? __________________________ |  | Yes/No | Yes/No | Yes/No  If no, how many______  Why? ______________________ |  |
| **Date** | Yes/No | Yes/No  If no, how many______  why? __________________________ |  | Yes/No | Yes/No | Yes/No  If no, how many______  Why? ______________________ |  |
| **Date** | Yes/No | Yes/No  If no, how many______  why? __________________________ |  | Yes/No | Yes/No | Yes/No  If no, how many______  Why? ______________________ |  |
| **Date** | Yes/No | Yes/No  If no, how many______  why? __________________________ |  | Yes/No | Yes/No | Yes/No  If no, how many______  Why? ______________________ |  |
| **Date** | Yes/No | Yes/No  If no, how many______  why? __________________________ |  | Yes/No | Yes/No | Yes/No  If no, how many______  Why? ______________________ |  |
| **Date** | Yes/No | Yes/No  If no, how many______  why? __________________________ |  | Yes/No | Yes/No | Yes/No  If no, how many______  Why? ______________________ |  |
| **Date** | Yes/No | Yes/No  If no, how many______  why? __________________________ |  | Yes/No | Yes/No | Yes/No  If no, how many______  Why? ______________________ |  |
| **Date** | Yes/No | Yes/No  If no, how many______  why? __________________________ |  | Yes/No | Yes/No | Yes/No  If no, how many______  Why? ______________________ |  |
| **Date** | Yes/No | Yes/No  If no, how many______  why? __________________________ |  | Yes/No | Yes/No | Yes/No  If no, how many______  Why? ______________________ |  |
| **Date** | Yes/No | Yes/No  If no, how many______  why? __________________________ |  | Yes/No | Yes/No | Yes/No  If no, how many______  Why? ______________________ |  |
| **Date** | Yes/No | Yes/No  If no, how many______  why? __________________________ |  | Yes/No | Yes/No | Yes/No  If no, how many______  Why? ______________________ |  |
| **Date** | Yes/No | Yes/No  If no, how many______  why? __________________________ |  | Yes/No | Yes/No | Yes/No  If no, how many______  Why? ______________________ |  |
| **Date** | Yes/No | Yes/No  If no, how many______  why? __________________________ |  | Yes/No | Yes/No | Yes/No  If no, how many______  Why? ______________________ |  |
| **Date** | Yes/No | Yes/No  If no, how many______  why? __________________________ |  | Yes/No | Yes/No | Yes/No  If no, how many______  Why? ______________________ |  |
| **Date** | Yes/No | Yes/No  If no, how many______  why? __________________________ |  | Yes/No | Yes/No | Yes/No  If no, how many______  Why? ______________________ |  |
| **Date** | Yes/No | Yes/No  If no, how many______  why? __________________________ |  | Yes/No | Yes/No | Yes/No  If no, how many______  Why? ______________________ |  |
| **Date** | Yes/No | Yes/No  If no, how many______  why? __________________________ |  | Yes/No | Yes/No | Yes/No  If no, how many______  Why? ______________________ |  |
| **Date** | Yes/No | Yes/No  If no, how many______  why? __________________________ |  | Yes/No | Yes/No | Yes/No  If no, how many______  Why? ______________________ |  |
| **Date** | Yes/No | Yes/No  If no, how many______  why? __________________________ |  | Yes/No | Yes/No | Yes/No  If no, how many______  Why? ______________________ |  |
| **Date** | Yes/No | Yes/No  If no, how many______  why? __________________________ |  | Yes/No | Yes/No | Yes/No  If no, how many______  Why? ______________________ |  |
| **Date** | Yes/No | Yes/No  If no, how many______  why? __________________________ |  | Yes/No | Yes/No | Yes/No  If no, how many______  Why? ______________________ |  |
| **Date** | Yes/No | Yes/No  If no, how many______  why? __________________________ |  | Yes/No | Yes/No | Yes/No  If no, how many______  Why? ______________________ |  |
| **Date** | Yes/No | Yes/No  If no, how many______  why? __________________________ |  | Yes/No | Yes/No | Yes/No  If no, how many______  Why? ______________________ |  |
| **Date** | Yes/No | Yes/No  If no, how many______  why? __________________________ |  | Yes/No | Yes/No | Yes/No  If no, how many______  Why? ______________________ |  |
| **Date** | Yes/No | Yes/No  If no, how many______  why? __________________________ |  | Yes/No | Yes/No | Yes/No  If no, how many______  Why? ______________________ |  |
| **Date** | Yes/No | Yes/No  If no, how many______  why? __________________________ |  | Yes/No | Yes/No | Yes/No  If no, how many______  Why? ______________________ |  |
| **Date** | Yes/No | Yes/No  If no, how many______  why? __________________________ |  | Yes/No | Yes/No | Yes/No  If no, how many______  Why? ______________________ |  |
| **Date** | Yes/No | Yes/No  If no, how many______  why? __________________________ |  | Yes/No | Yes/No | Yes/No  If no, how many______  Why? ______________________ |  |
| **Date** | Yes/No | Yes/No  If no, how many______  why? __________________________ |  | Yes/No | Yes/No | Yes/No  If no, how many______  Why? ______________________ |  |
| **Date** | Yes/No | Yes/No  If no, how many______  why? __________________________ |  | Yes/No | Yes/No | Yes/No  If no, how many______  Why? ______________________ |  |
| **Date** | Yes/No | Yes/No  If no, how many______  why? __________________________ |  | Yes/No | Yes/No | Yes/No  If no, how many______  Why? ______________________ |  |
| **Date** | Yes/No | Yes/No  If no, how many______  why? __________________________ |  | Yes/No | Yes/No | Yes/No  If no, how many______  Why? ______________________ |  |
