# Supplementary figures and images for: Does radial shockwave therapy lead to immediate improvements in pain in people with insertional Achilles tendinopathy? A randomised controlled trial
Source: Clin Rehabil. 2025 Nov 27;40(2):171–81. doi: 10.1177/02692155251394951 (PMC12816402; doi:10.1177/02692155251394951)

**Supplementary File 3:** Description of straight and bent knee exercises


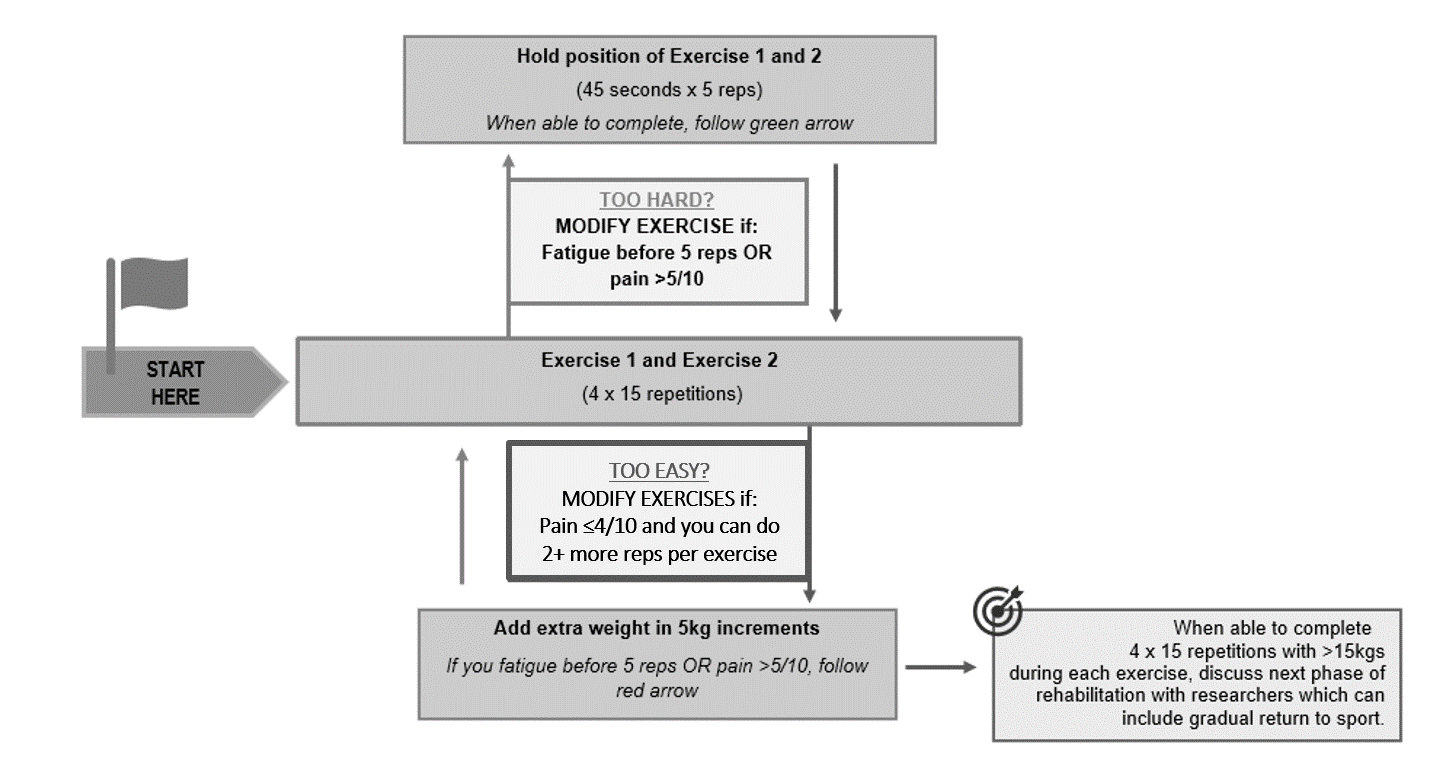

Supplement: sj-docx-3-cre-10.1177_02692155251394951 - Supplemental material for Does radial shockwave therapy lead to immediate improvements in pain in people with insertional Achilles tendinopathy? A randomised controlled trial [file sj-docx-3-cre-10.1177_02692155251394951.docx]
