# Supplementary material for: Does radial shockwave therapy lead to immediate improvements in pain in people with insertional Achilles tendinopathy? A randomised controlled trial
Source: Clin Rehabil. 2025 Nov 27;40(2):171–81. doi: 10.1177/02692155251394951 (PMC12816402; doi:10.1177/02692155251394951)
Supplement: sj-docx-4-cre-10.1177_02692155251394951 - Supplemental material for Does radial shockwave therapy lead to immediate improvements in pain in people with insertional Achilles tendinopathy? A randomised controlled trial [file sj-docx-4-cre-10.1177_02692155251394951.docx]

**Supplementary File 4:** Participant’s beliefs about authenticity of the intervention and sham

| **Week** | **Radial shockwave** | | **Sham** | | **Chi-square** |
| --- | --- | --- | --- | --- | --- |
|  | **Real** | **Unsure** | **Real** | **Unsure** |  |
| **1** | 65.8% | 34.2% | 47.4% | 47.4% | 0.17 |
| **2** | 63.2% | 31.6% | 44.7% | 39.5% | 0.26 |
| **3** | 73.7% | 26.3% | 52.6% | 39.5% | 0.14 |
